# Integrating Traditional Chinese Medicine Services in Community Health Centers: Insights into Utilization Patterns in the Pearl River Region of China

**DOI:** 10.1155/2013/426360

**Published:** 2013-02-20

**Authors:** Vincent C. H. Chung, Polly H. X. Ma, Harry H. X. Wang, Jia Ji Wang, Lau Chun Hong, Xiaolin Wei, Samuel Y. S. Wong, Jin Ling Tang, Sian M. Griffiths

**Affiliations:** ^1^Jockey Club School of Public Health and Primary Care, Chinese University of Hong Kong, Hong Kong; ^2^School of Public Health, Guangzhou Medical University, Guangzhou, China

## Abstract

In China's healthcare reform, community health centers (CHCs) are designed to take a pivotal role in providing primary care. Whilst about 20% of all outpatient care in China is delivered by the traditional Chinese medicine (TCM) sector, hospitals, instead of CHCs, are major providers. Using current patterns of patient utilization this study aims to inform CHCs on how they may strengthen access to TCM services. Three thousand three hundred and sixty CHC patients from six cities within the urban Pearl Delta Region were enumerated using multistage cluster sampling. Fifty-two percent had visited herbalists within three months with a mean visit frequency of 1.50 times. Herbal treatments, which are cheaper than western medicines, were more popular amongst those who needed to pay out of pocket including the uninsured. Herbal medicines appeared to be an alternative for those who are underinsured. Acupuncturists and massage therapists were visited by smaller proportions, 6.58% and 5.98%, respectively, with a mean three-month visit of 0.27 and 0.26 times. Access was restricted by lack of social insurance coverage. Whilst increasing provision of TCM in CHCs might respond to patient demand, increasing insurance coverage for TCM needs to be evaluated using current evidence on safety and effectiveness.

## 1. Introduction

### 1.1. Primary Care as the Cornerstone of Healthcare Reform in China

Strengthening primary care as a foundation and entry point of the healthcare system is the cornerstone of China's healthcare reform. Current reform proposals aim to position community health services as major providers of first-line care for common clinical problems [1]. Across China, the number of community health service facilities increased by threefold during 2001 to 2008 [1]. By 2010, China had established 33,000 community health services organizations across the nation, with some 29,500 employees [2, 3]. Amongst these organizations, Community Health Centers (CHCs) have been established and are usually buildings with areas of more than 1,000 square meters and with fewer than 50 inpatient beds. CHCs are purposefully established for providing medical and preventive outpatient services to a population of 30,000–50,000 [4]. Together with conventional biomedicine (BM), traditional Chinese medicine (TCM) is a formally recognized part of the Chinese healthcare system [5], and the central government has listed TCM as a required service in all CHCs [6].

### 1.2. Contribution of Traditional Chinese Medicine in Primary Care Service Provision

According to a 2004 national survey that covered both urban and rural China, 14% of the enumerated households identified TCM as their typical source of care [7]. This figure is consistent with the 2006 official data, which estimated that 10–20% of all healthcare services in China were provided by the TCM sector [8]. If only outpatient services were counted, the figure would fall in the higher end of this range. A large scale survey of 739,600 healthcare organizations undertaken in China in 2009 [9] reported that 19.2% of all outpatient consultations were managed by TCM clinicians, which translates into 0.67 billion visits/year. Surprisingly, as little as 5.1% of this enormous volume of visits were to CHCs [9]. 

In general, distrust in the quality of care is a possible reason for deterring patients from choosing CHC services [10, 11]. In a recent patient satisfaction survey in Dalian, 91% of users had low trust in doctors working in CHCs, and 75% had no confidence in the quality of their services [12]. Another large scale survey found that only 35% of patients consider CHCs as a safe source of care [1]. Hence, hospitals remain to be the major providers of TCM outpatient services [13], instead of CHCs. However, under the context of privatization since 1990s, incentives to provide TCM care in hospitals are decreasing as herbal medicine prescriptions, acupuncture and massage therapies are not considered to be “revenue generating” in secondary care settings. TCM clinicians are inclined to replace their traditional practice with BM treatments [14]. As a result, there is a greater potential of promoting TCM in CHC settings. 

### 1.3. Strengthening Traditional Chinese Medicine Services in Community Health Centers

To redress the mismatch of TCM underutilization in CHCs and overutilization in hospitals, top down policies from the central government have been promulgated. In 2006, the Ministry of Health and the State Administration of Traditional Chinese medicine jointly announced a mandate that each CHC must have at least one clinician specializing in TCM, together with a herbal pharmacy and equipment support [15]. Nevertheless, statistics from 2009 showed that only 51.6% of the nation's CHCs possessed the infrastructure for TCM service provision [9], and only 22% of the CHC clinicians offered TCM treatment [1]. Consequent on this lack of progress, improving the role of TCM in enhancing the nation's basic healthcare system has been restated as a goal in the national 12th Five-Year Plan and is also listed as one of the top 10 national health priorities in 2011 [16, 17]. If implemented successfully, the capacity of CHCs in providing TCM services would be enhanced significantly in the future. However, regardless of service location, TCM outpatient services are not fully covered by the three major healthcare insurance schemes in China, namely, New Rural Cooperative Medical Scheme (NCMS); Urban Employee Basic Medical Insurance (UEBMI); and Urban Residents Basic Medical Insurance (URBMI) [18]. It is however covered by the government sponsored care scheme [19]. 

The Pearl River Delta (PDR) region of the urban Guangdong province is an economic power house of China. It consists of six major cities: Shenzhen, Guangzhou, Foshan, Zhongshan, Zhuhai, and Dongguan, with a total of nearly 1000 CHCs providing care for a population of about 43.2 million, many of whom are migrant workers. Consistent with the central directives, the Guangdong government is keen to strengthen TCM services amongst CHCs within the PDR region [20]. In order to inform service redesign, a deeper understanding of the characteristics and health seeking pattern of patients who choose to use TCM services in the community is needed. This will allow the design of appropriate improvement strategies that are responsive to patients' choice.

### 1.4. Aim of the Study

This study described the utilization of TCM services by those attending CHCs. We investigated the (i) prevalence and frequency of TCM use as well as the (ii) demographic and health-related characteristics of TCM users as compared to those who only utilize BM services. In addition, we also examine the patients' (iii) reason for consultation and their perceived effectiveness of TCM treatments. 

## 2. Methods

### 2.1. Sampling and Data Collection

CHCs for inclusion in the study were selected using multistage cluster sampling in six major cities in the Pearl River Delta: Guangzhou, Shenzhen, Dongguan, Zhuhai, Foshan, and Zhongshan. In the 1st stage, 4 districts in each city were randomly selected. In the 2nd stage, 1 neighborhood in each of the 4 districts was randomly selected. In the 3rd stage, one CHC was selected in each of the 4 neighborhoods. We estimated a total sample size requirement of 3360, which was calculated in accordance with the requirement for conducting multivariate analyses [21]. For each city except Guangzhou, data collection continued until the sample size of 480 was reached. Given its larger population and geographical size, we collected data from Guangzhou until a larger sample size of 960 was achieved. 

In each CHC, all service users aged ≥18 were invited to participate in a face-to-face interview during the opening hours. We invited all patients who attended the CHCs until the required sample size was reached. A cash incentive of RMB $25 was offered to all participants who completed the questionnaire. Written informed consent was obtained from patients prior to the interview. Ethics approval was obtained from the Survey and Behavioral Research Ethics Committee of the Chinese University of Hong Kong. 

### 2.2. Questionnaire Design

The questionnaire used in the interview consisted of two parts. The first part aimed to collect data on the respondents' demographic and health-related characteristics, including their gender, age, household registry status (*Hukou*), education level, household income, occupation, insurance status, self-perceived health status, and chronic disease status. In the second part, we assessed the use of BM and TCM using a modified International Complementary and Alternative Medicine Questionnaire (I-CAM-Q) [22]. Specifically, respondents were asked to indicate whether they had visited the following types of clinicians in the past 12 months, regardless of location: (i) western trained biomedical doctors (BMD); (ii) TCM herbalists; (iii) TCM acupuncturists; and (iv) TCM massage (Tuina) therapists. If the respondents provided a positive response, they were asked to specify consultation frequency in the past 3 months. Moreover, they were invited to indicate the main reason for visiting in the latest consultation. Four options were provided: (i) for acute condition that lasted <1 month; (ii) for chronic condition that lasted ≥1 month; and (iii) for improving well-being. Finally, they were asked to evaluate treatment effectiveness on a scale including very helpful, somewhat helpful, not helpful at all, and do not know.

### 2.3. Data Analysis

Prevalence and its 95% confidence interval (CI) of past year consultation was reported for each type of clinician, together with their corresponding frequency of visits in the past three months. Multiple logistic regression analyses were conducted to identify demographic and health-related characteristics related to TCM use, with reference to those who only consulted BMD in the past year. A separate multiple logistic regression analysis was conducted for each of the modalities. Proportions and 95% CIs of main reason for consultation (visiting for acute conditions, chronic conditions, and well-being improvement) were calculated. Chi-square goodness of fit test was performed to test the equality of frequencies among different reasons for each type of consultation. For significant results of Chi-square goodness of fit test, post hoc one-sample Chi-square tests were conducted for pairwise comparison of two categories by setting the hypothesized frequencies of other categories as the observed frequencies. In addition, the proportions of self-reported treatment effectiveness were calculated together with their respective 95% CIs. 

## 3. Results

### 3.1. Prevalence and Frequency TCM Service Use amongst CHCs Patients

Three thousand three hundred and sixty patients were interviewed within the prespecified quota from all six cities. The overall response rate was 86.1%.  Table 1 displays the demographic and health-related characteristics of our respondents. In the past 12 months, prevalence of consulting BMD at least once is 91.37% (95%CI: 91.36%, 91.37%), with a mean three-month visit frequency of 3.35 (SD = 5.72). The prevalence for visiting a herbalist in the past year is 51.70% (95%CI: 51.68%, 51.71%), with a mean three-month visit frequency of 1.50 (SD = 3.62). Acupuncturists and massage therapists were visited by a smaller proportion of 6.58% (95%CI: 6.57%, 6.58%) and 5.98% (95%CI: 5.98%, 5.99%). The mean three-month visit frequencies for the two modalities were 0.27 (SD = 1.95) and 0.26 (SD = 1.69), respectively. 

### 3.2. Demographic and Health-Related Characteristics of TCM Users

Compared to respondents who were financially covered by government sponsorship, multiple logistic regression analyses (Table 2) showed that those who paid by other means, including UEBMI, URBMI, NCMS, and out of pocket, were less likely to consult acupuncturists or massage therapists. Interestingly, those who paid out of pocket were more likely to use herbal medicine services, compared to those who were entitled to government sponsored care. Those who rated their health status as poor were also less likely to use acupuncture services compared to those who perceived to be in good health. Older respondents were more likely to consult herbalists, and those who completed secondary education were less likely. In all three multiple logistic regression analyses, we have more than 10 utilization events per independent variable; thus our sample size is sufficient for conducting such analyses [21]. 

### 3.3. Main Reason for Consultation and Perceived Effectiveness

**Figure 1:**
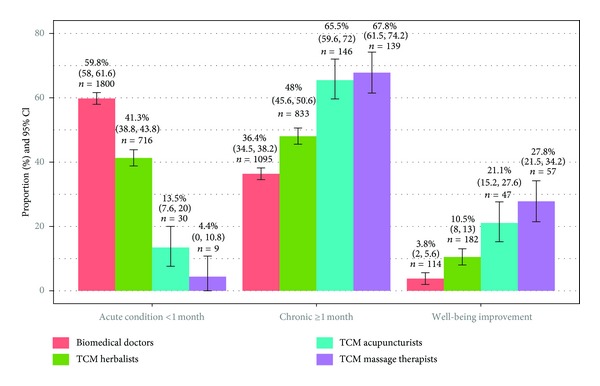
Main reason for visiting in the latest consultation, stratified by treatment modalities.

**Figure 2:**
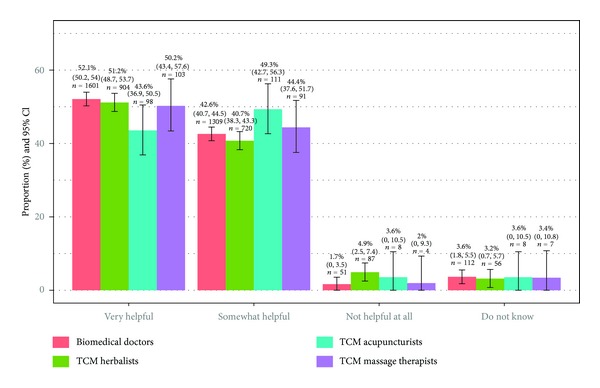
Perceived effectiveness of treatment received in the latest consultation episode, stratified by modalities.

Figure 1 shows the main reasons for consultation for the most recent visit within the past twelve months, stratified by treatment modalities. For all modalities, the null hypotheses of Chi-square goodness of fit tests (all categories of main reason for consultation occur with equal probabilities) were rejected at *P* < 0.001. Majority consulted BMD for treatment of acute conditions, which is a significantly higher proportion (*P* < 0.001) compared to patients visiting for chronic conditions. A very low proportion of patients consulted a BMD for improving well-being. The inverse pattern was observed for massage therapy. A very low proportion received massage for acute conditions, and the figure is significantly (*P* < 0.001) lower than the proportions of patients who visited for chronic condition or well-being improvement. The distribution for acupuncture was similar. Compared with patients seeking help for their chronic conditions, proportion of those consulting acupuncturists for acute conditions is significantly lower (*P* < 0.001); but not the proportion seeking improvement in wellbeing (*P* = 0.153). Although TCM herbalists appeared to have a more balanced patient profile, proportion of consultation for acute conditions is significantly (*P* = 0.032) lower than that for chronic condition. A much lower proportion consulted herbalists for well-being improvement. Regardless of modality, the majority of patients found the treatment very helpful or somewhat helpful (Figure 2).

## 4. Discussion

Our results indicated that not only is provision of TCM a government policy but also it is a popular choice for patients, used by many CHC attendees. Other studies have found that patients purposefully chose these modalities to fill the perceived effectiveness gaps of BM [23]. Herbal medicine, acupuncture, and massage therapies are preferred in the treatment of chronic conditions which have lasted for more than 1 month, while BM remained to be the most popular option for acute conditions. More than 20% of acupuncture and massage service users sought to improve well-being, but only very low proportion of visits to western trained doctors was for this purpose. This is a common pattern and the challenge to policy makers and service providers is to develop services which reflect the different choices patients make for services at different stages of ill health. This does, however, raise interesting questions about the evidence of effectiveness between modalities and the influence of affordability in the trade-offs made by patients. 

### 4.1. Chinese Herbal Medicine Services as an Accessible Form of Care for the Uninsured or Underinsured

Chinese herbal medicine appeared to be the most popular form of TCM amongst those attending CHCs. More than half had consulted a TCM herbalist in the past year. This is consistent with previous studies which have found that herbal medicine services are more popular amongst older segment of the population, as well as the better or poorer educated [7, 24]. Since in China a qualified TCM herbalist can prescribe both conventional western drugs as well as herbs in a single consultation [14], users of herbalist services included both acute and chronic patients. This contrasts with findings from the West where herbalists are often consulted to complement BM treatment amongst chronic disease patients [25]. 

Of note, herbalist service appeared to be more popular amongst those who needed to pay out of pocket, which includes those who have no or minimal outpatient insurance coverage and who choose to use herbal medicine as an alternative to more expensive BM drugs. The inclusion of Chinese herbal medicines under the 2009 National Essential and Health Insurance Drug Lists has made them financially affordable to most patients. Amongst the 307 medications on the Chinese National Essential Drug List, 102 are herbal products and they are sold at the guaranteed lowest price. The National Health Insurance Drug List has also included a total of 683 herbal medications, of which the maximum co-payment is only 10% [13, 19]. 

### 4.2. Financial Barriers Hinder Access to Acupuncture and Massage Services

In contrast to herbal medicine, prevalence of acupuncture and massage service use is much lower. CHC attendees seem to reserve these options for the treatment of chronic conditions or well-being improvement, instead of using them for managing acute conditions. This pattern is consistent with previous studies in China, where acupuncture was perceived to have special strength in certain chronic disorders [26] and was less useful in managing common ailments [27]. Also, it is believed that patients with poorer health are more susceptible to adverse effects of acupuncture [28]. This may explain why respondents with poor self-rated health are less likely to consult an acupuncturist. 

In addition to patients' perception of the appropriateness and effectiveness of the TCM treatment, financial barriers offer additional explanation for lower utilization rates of acupuncture and massage services. Our results demonstrated that those who possess NCMS, UEBMI, and URBMI are less likely to use these services, as the reimbursement mechanisms of these plans often fail to cover treatment costs. In the majority of NCMS and UEBMI plans, outpatient services are often paid through personal medical saving accounts (MSA), and patients are expected to pay out of pocket when the MSA fund is exhausted. For URBMI, pooled funds only cover outpatient services for targeted chronic or catastrophic diseases [29]. The financial pressure of paying out of pocket for acupuncture and massage services, which often require multiple sessions of treatment, may have deterred patients from using them. 

### 4.3. Strengths and Weaknesses of This Study

The most notable strength of this study is that we avoided selection bias by using a multistage cluster sampling strategy. This allowed us to draw a representative sample of CHCs attendees in all six major cities within the urban PDR region. Meanwhile, our study has a number of limitations. First, cross-sectional design has inhibited us from drawing a causal conclusion. A cohort study is needed to ascertain whether the associations observed between TCM use and various health and demographic factors are causal. Second, CHCs attendees were asked to recall the outpatient service utilization and thus recall bias could have led to inflation or deflation of visit frequencies. The use of pretested I-CAM-Q items has allowed us to collect standardized utilization data that allows international comparison [30]. It has demonstrated strong face validity and acceptability in our sample but formal assessment on the Chinese version's reliability and validity is needed in the future. Finally, although we achieved a high response rate, we were unable to assess the potential impact of nonresponse bias due to a lack of sampling frames that contain background information of nonrespondents. 

### 4.4. Further Research

Policy makers in China are adopting strategies to facilitate the channeling of TCM patients from hospitals to CHCs. Encouraging current hospital attendees to use locally based TCM services could be a first step in moving patients to the community. Future studies are needed to compare TCM users' profiles in CHCs and outpatient departments of hospitals and investigate factors that facilitate or hinder the use of CHCs based TCM services. In a similar vein, studies that examine why some patients only use BM, but not TCM services in CHCs settings is also warranted. Finally, future investigations using qualitative method is needed to triangulate our current quantitative findings. For example, focus groups or in-depth interviews maybe conducted for exploring how patients managed the impact of different financial barriers on the use of different TCM modalities.

## 5. Conclusion

TCM is popular amongst CHCs attendees in the urban PDR region, especially services provided by herbalists. Due to the lower cost of herbal medicines, such service may be regarded an alternative treatment of choice for those who need to pay out of pocket. CHCs in the region may have the potential of increasing herbal medicine use by up-scaling their herbalist services. However, since the efficacy and safety of many Chinese herbal medicines remains to be uncertain [31], evidence-based decision making in this area is a complex problem. Large scale randomized controlled trials of herbal medicines included in the Essential and Health Insurance Drug Lists should be given research priority to enable better understanding of on their efficacy and safety, either prescribed alone or with allopathic drugs [32]. On the other hand, while acupuncture and massage appear to have a relatively stronger evidence base supporting their efficacy and safety [33, 34], their access is limited by financial barriers. This implies that simply strengthening provision of these services at CHCs may not increase utilization until NCMS, UEBMI, and URBMI extend their coverage on outpatient services. In the longer term, decisions on planning and funding of TCM services in CHCs should be made after considering clinical evidence, cost effectiveness, and patients' choice. 

## Figures and Tables

**Table 1:** Demographic and health-related characteristics of respondents.

Characteristics	Number of respondents
	(%)
Gender	
Male	1423 (42.4%)
Female	1933 (57.6%)
Household registry^†^	
Resident with Hukou	1726 (51.4%)
Resident without Hukou	1284 (38.2%)
Nonresident without Hukou	349 (10.4%)
Education level	
Tertiary education or above	828 (24.7%)
Secondary education	1975 (58.9%)
Primary education or below	551 (16.4%)
Household income (¥)	
<1000	272 (9.9%)
1000–2000	903 (33.0%)
2001–3000	708 (25.9%)
3001–4000	333 (12.2%)
4001–5000	235 (8.6%)
>5000	285 (10.4%)
Insurance status^#^	
Government sponsored medical care	249 (7.5%)
UEBMI	905 (27.1%)
URBMI	707 (21.2%)
NCMS	470 (14.1%)
Commercial insurance	22 (0.7%)
Out of pocket	982 (29.4%)
Self-perceived health status	
Excellent	207 (6.2%)
Very good	735 (21.9%)
Good	886 (26.4%)
Fair	1378 (41.0%)
Poor	152 (4.5%)
No. of chronic diseases	
0	2194 (65.3%)
1	830 (24.7%)
2	239 (7.1%)
3	83 (2.5%)
4	12 (0.4%)
5	2 (0.1%)
Mean age (SD)*	43.4 (17.05)

*Data are presented as mean (SD).

^†^Hukou: household registration; resident with hukou: permanent resident. Resident without hukou: temporary resident without local household registration but lived in the city ≥6 months. Nonresident without hukou: temporary resident without local household registration and lived in the city <6 months.

^#^UEBMI: urban employee basic medical insurance; URBMI: urban resident basic medical insurance; NCMS: new cooperative medical scheme.

**Table 2:** Association between TCM usage and demographic and health characteristics: multiple logistic regression analyses.

TCM modalities	Herbalists		Acupuncturists		Massage therapists	
Demographic characteristics	Adjusted OR (95% CI)	*P* value	Adjusted OR (95% CI)	*P* value	Adjusted OR (95% CI)	*P* value
Gender						
Male (reference)	1.000		1.000		1.000	
Female	1.155 (.988, 1.351)	.070	.822 (.603, 1.119)	.213	.936 (.676, 1.297)	.692
Age	1.007 (1.000, 1.014)	.039	1.008 (.995, 1.021)	.255	1.007 (.993, 1.020)	.348
Hukou status						
Resident with Hukou (reference)	1.000		1.000		1.000	
Resident without Hukou	.870 (.714, 1.061)	.169	.985 (.657, 1.478)	.943	.851 (.551, 1.315)	.468
Non-Resident without Hukou	.829 (.614, 1.119)	.221	1.432 (.807, 2.540)	.219	.787 (.388, 1.594)	.505
Education level						
Tertiary education (reference)	1.000		1.000		1.000	
Secondary education	.764 (.622, .940)	.011	.820 (.550, 1.223)	.331	.802 (.532, 1.209)	.292
Primary education	.783 (.575, 1.066)	.121	.637 (.341, 1.190)	.157	.550 (.284, 1.066)	.077
Monthly household income	1.005 (.947, 1.067)	.860	1.091 (.967, 1.232)	.157	1.040 (.919, 1.178)	.534
Health insurance status						
Government sponsored care (reference)	1.000		1.000		1.000	
Out of pocket	1.507 (1.045, 2.174)	.028	.411 (.215, .786)	.007	.333 (.176, .631)	.001
Urban employee basic medical insurance	1.127 (.811, 1.567)	.476	.506 (.291, .881)	.016	.356 (.210, .601)	.000
Urban resident basic medical insurance	1.063 (.748, 1.509)	.734	.542 (.297, .987)	.045	.356 (.198, .641)	.001
New cooperative medical scheme	1.119 (.764, 1.637)	.565	.498 (.253, .982)	.044	.210 (.098, .452)	.000
Self-reported health status						
Good or above (reference)	1.000		1.000		1.000	
Fair	1.020 (.864, 1.203)	.817	.959 (.693, 1.327)	.802	1.189 (.843, 1.676)	.323
Poor	1.087 (.731, 1.617)	.680	.196 (.047, .824)	.026	.701 (.284, 1.734)	.442
Number of chronic disease						
0 (reference)	1.000		1.000		1.000	
1	1.174 (.960, 1.436)	.119	1.092 (.739, 1.614)	.658	.927 (.605, 1.422)	.730
2	1.004 (.715, 1.410)	.982	.692 (.333, 1.436)	.323	.904 (.463, 1.766)	.767
≥3	1.178 (.728, 1.907)	.505	1.116 (.470, 2.648)	.804	1.533 (.717, 3.279)	.271
